# Improvement of Adhesion Properties and Corrosion Resistance of Sol-Gel Coating on Zinc

**DOI:** 10.3390/molecules23051079

**Published:** 2018-05-03

**Authors:** Pauline Savignac, Marie-Joëlle Menu, Marie Gressier, Bastien Denat, Yacine El Khadir, Stephan Manov, Florence Ansart

**Affiliations:** 1 VM Building Solutions, Les Mercuriales, Tour du Ponant, 40 rue Jean Jaurès, 93170 Bagnolet, France; bastien.denat@gmail.com (B.D.); elkhadir.yacine@gmx.fr (Y.E.K.); stephan.manov@vmzinc.com (S.M.); 2 CIRIMAT, Université de Toulouse, CNRS INPT UPS, UMR 5085, 118 Route de Narbonne, 31062 Toulouse CEDEX 09, France; menu@chimie.ups-tlse.fr (M.-J.M.); gressier@chimie.ups-tlse.fr (M.G.); ansart@chimie.ups-tlse.fr (F.A.)

**Keywords:** sol-gel, hybrid materials, corrosion, adhesion, coating, zinc

## Abstract

Corrosion is a major problem for durability of many metals and alloys. Among the efficient classical surface treatments, chromate-based treatments must be banished from industrial use due to their toxicity. At the same time, sol-gel routes have demonstrated high potential to develop an efficient barrier effect against aggressive environments. By this process, the anti-corrosion property can be also associated to others in the case of the development of multi-functional hybrid coatings. In this paper, the main goal is precisely to improve both the corrosion resistance and the adhesion properties of phosphated zinc substrates by the deposition of a hybrid (organic-inorganic) sol-gel layer. To reach this double objective, a choice between two formulations 3-glycidoxypropyltrimethoxysilane (GPTMS)/aluminum-tri-sec-butoxide (ASB) and 3-(trimethoxysilyl)propylmethacrylate (MAP)/tetraethylorthosilicate (TEOS) was firstly made based on the results obtained by microstructural characterizations using SEM, optical analysis, and mechanical characterization such as shock and/or scratch tests (coupled to climatic chamber and salt spray exposure). Several investigations were performed in this study, and the best formulation and performances of the system were obtained by adding a new precursor (1-[3-(trimethoxysilyl)propyl]ureido-UPS) under controlled conditions, as detailed in this paper.

## 1. Introduction

Corrosion protection of metallic substrates using coatings is an active and important research area in materials science and engineering. Most metals need to be protected against corrosion to slow down and limit the effects of aging and degradation. Zinc is a material used in many fields for its own characteristics: durability, ease of shaping, ductility or low corrosion potential. Due to this latter property, it is used to provide a protective layer to steel by galvanizing processes, where it plays the role of sacrificial anode.

Its ability to naturally create a patina layer (complex compound mainly containing zinc hydroxycardonates), protecting it from external elements [1] and avoiding the formation of corrosion products, is one of its main advantages. However, for some applications, it is still necessary to provide a protective layer to further improve its resistance to corrosion. The requirement to replace the harmful chromate-based surface treatments with environmentally friendly methods has for many years promoted the development of new treatments for different kinds of substrates. Organic-inorganic hybrid sol-gel coatings are considered a promising alternative due to their easy preparation at low temperature, their low cost, and their versatility of deposition on many metallic substrates [2,3,4,5], including aluminum alloys [6,7], steel alloys [7,8] and zinc [4,9,10,11,12].

Nevertheless, before the development of the sol-gel process, chromium-free chemical conversion treatments were investigated to tackle the corrosion phenomenon; such as phosphatizing, especially on zinc and zinc-coated steel, making it possible to create a layer of zinc phosphates more resistant to corrosion on the surface of the metal. The results presented here were carried out on phosphated zinc, and even if the surface of the galvanized steel is zinc, the behavior of the material could be different, if we consider the corrosion behavior as shown by the electrochemistry studies of Eliziane [8]. This is why it is not always obvious to extrapolate the results obtained on galvanized steel to protect zinc and vice versa [13,14,15].

Despite this initial protection, species contained in the atmosphere (such as chlorides or sulfides) can damage this “protective” layer and lead to the formation of white rust. Therefore, the deposition of an additional coating is necessary. The development of sol-gel processes has made it possible to provide an efficient coating against corrosion for metals in various fields, particularly in aeronautics, as can be seen in the literature on this topic, which has enhanced the performance of other substrates used in other fields [4]. New regulations and improvements in know-how have led to the development of sol-gel coating processes for metal protection, and particularly for zinc, in this case. Organic–inorganic sol-gel coatings can improve corrosion resistance, and in addition, other properties can be also provided by the sol-gel layer. Indeed, many publications on sol-gel coating deal with galvanized steel substrates, but few employ zinc as a working substrate [4,13,16].

**Figure 1:**
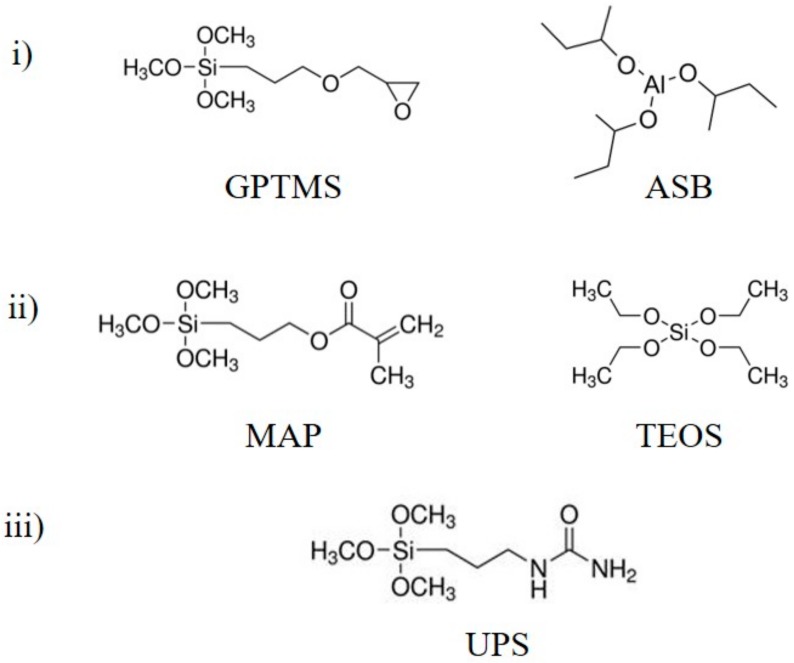
Chemical structures of organic and inorganic network precursors: (**i**) GPTMS and ASB, (**ii**) MAP and TEOS, and (**iii**) UPS.

In our case, efficient protections against corrosion associated with good adhesion properties are researched directly on phosphated zinc for applications mainly in the building sector. Commonly, sol-gel processes involve alkoxysilane precursors as starting materials. In this study, two sol-gel formulations were first investigated based on previous work developed on aluminum alloys [17] or stainless steel [18], as well as literature [4]: (i) 3-glycidoxypropyltrimethoxysilane (GPTMS)/aluminum-tri-sec-butoxide (ASB), and (ii) 3-(trimethoxysilyl)propylmethacrylate (MAP)/tetraethylorthosilicate (TEOS), whose chemical structures are shown in Figure 1. In both formulations, the precursors contributed to creating the organic and inorganic parts to provide organoaluminosilicated (i) and organosilicated (ii) hybrid coatings, respectively. These precursors are mixed in alcohol and water, and cerium (III) nitrate hexahydrate is systematically added as an eco-friendly corrosion inhibitor in the optimized concentration of 0.01 mol L^−1^ [16,17,18]. The behavior of the coated samples is evaluated, especially the corrosion resistance with accelerated corrosion testing (climatic chamber and salt spray) and the adhesive properties by mechanical tests (shock test and scratch test). This work proposes an optimization of the most efficient formulations by adding an ureido precursor 1-[3-(trimethoxysilyl)propyl]ureido (UPS) (Figure 1iii). This ureido precursor has been chosen to increase the chemical compatibility with the polyurethane top-coat paint applied on zinc substrates. It should be noted that bis[(ureapropyl)triethosilyl groups are introduced in sol-gel coating to obtain a hydrophobic urea-polydimethylsiloxane sol-gel coating on aluminum alloys [19,20]. In these reported works, the bis-urea derivative is introduced in an amount of less than 4% wt in the sol-gel network. Introduction of such precursors constitutes an innovative sol-gel formulation on natural or phosphated zinc substrates, which could be developed on many other metal substrates.

The coatings are characterized by FT-IR and scanning electron microscopy (FEG-SEM) to determine the correlation between the sol formulation and the working properties.

Moreover, one of the innovative points here is that the experiments were carried out on phosphated zinc, by a chemical conversion process.

## 2. Results and Discussion

### 2.1. TEOS/MAP and GPTMS/ASB Coatings

**Figure 2:**
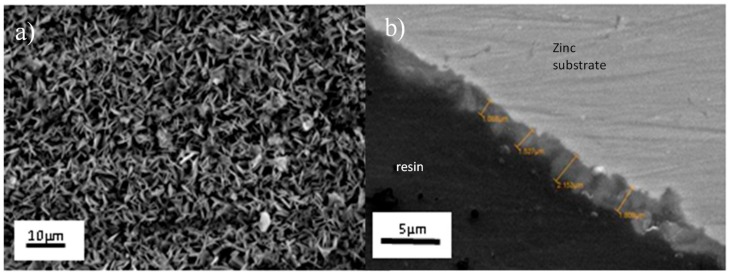
Scanning electron micrographs of phosphated zinc of (**a**) surface, and (**b**) cross-section.

The substrate consists of a degreased and cleaned natural zinc on which a phosphatation chemical conversion layer is deposited. After this step, it looks like a naturally-aged patina, with its typical matt-grey color. After surface treatment, the zinc is covered with phosphate crystals less than 2 µm in size (Figure 2a), which initially improves the corrosion resistance. The thickness of the layer is around 1.5–2 µm, and gives it a surface roughness of 1 µm corresponding to a layer of crystals as showed in Figure 2b.

**Figure 3:**
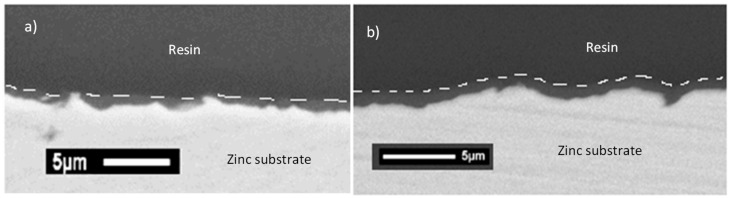
Scanning electron micrographs of cross-section of coatings deposited on natural zinc: (**a**) TEOS/MAP, and (**b**) GPTMS/ASB.

TEOS/MAP- and GPTMS/ASB-based coatings are deposited on phosphated zinc substrates by a dip-coating process. In parallel, in order to determine the layer thicknesses, the two sol-gel formulations were directly applied on natural zinc substrates (surface roughness around 0.8 µm). In fact, due to the microstructure of phosphated zinc surface with phosphate crystals resulting in a roughness of 1 µm, it is more difficult to evaluate the real coating thickness. Figure 3 shows that the coatings are conformal, covering, and levelling, and the thickness is 0.5 µm for the TEOS/MAP coating and 0.8 µm for the GPTMS/ASB coating on zinc substrate. The mass gains of deposits are around 10 mg/dm^2^ and 45 mg/dm^2^ for coatings of TEOS/MAP and GPTMS/ASB, respectively, yielding a density of the GPTMS/ASB coating that is 2.8 times higher than that of the TEOS/MAP coating.

These low coating thicknesses will not be able to completely cover the roughness of phosphated zinc, which is of the same order of magnitude as the deposit thickness. Nevertheless, even if the thickness of the coating is not enough to completely cover the phosphate crystals at the surface of the substrate, tests of corrosion and adhesion properties were conducted.

**Figure 4:**
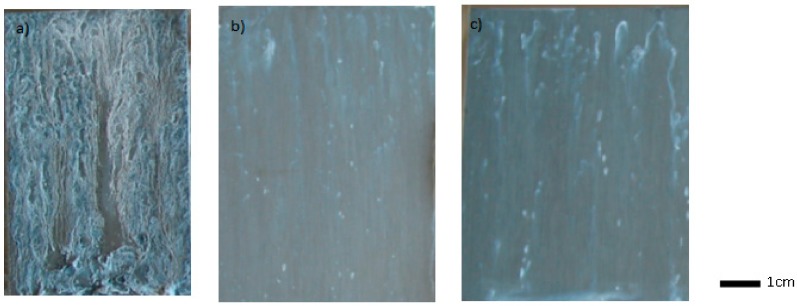
Salt spray test performed on (**a**) phosphated zinc for 24 h; (**b**) phosphated zinc + TEOS/MAP coating after 96 h; and (**c**) phosphated zinc + GPTMS/ASB coating after 96 h.

While phosphated zinc is completely degraded after 24 h in the salt spray chamber (Figure 4a), the samples coated with sol-gel films show slight marks of corrosion after 96 h of testing (Figure 4b,c). The presence of the sol-gel coating significantly improves the corrosion resistance, even if the covering is not optimized due to the surface topography. A better surface coverage of the phosphate crystals on the zinc substrates could further enhance this property.

**Figure 5:**
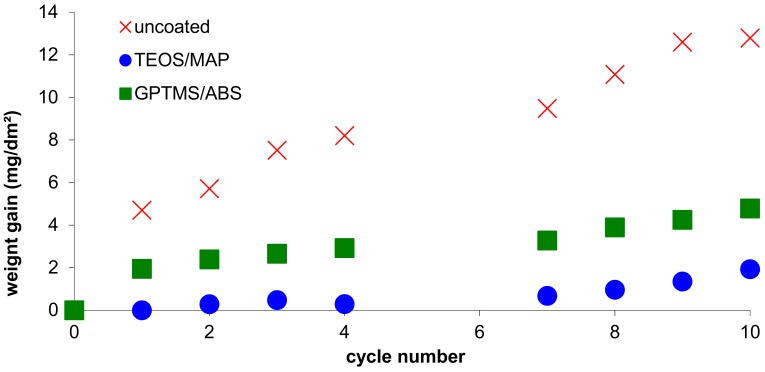
Weight gain during climatic chamber test.

Tests in a climatic chamber, as well as a salt spray test, were performed. The uncoated sample quickly degrades with the presence of white rust over the surface, and a much larger weight gain is measured compared to the sol-gel coated samples (Figure 5). Although no visual difference was observed between the 2 coatings after 96 h of salt spray test and 10 cycles in the climatic chamber, there was a difference between the behavior of the samples when comparing their weight gain, as shown in Figure 4. The weight gain after 10 cycles was about 1.9 mg/m^2^ for the TEOS/MAP coating, whereas it was about 4.8 mg/m^2^ for the GPTMS/ABS coating, indicating that this second formulation is twice as sensitive to corrosion as the first one for an equivalent coating thickness. Despite the higher density of the GPTMS/ABS coating, the anti-corrosion barrier provided is not sufficient.

From an industrial point of view, to characterize the adhesion properties of the sol-gel coating, a layer of polyurethane paint (black color) is applied to the substrate (phosphated zinc + sol-gel coating), making the determination of the adhesion performances of the sol-gel coating easier and more reproducible. This evaluation method was carried out because, at an industrial scale, the reference is a system including this top-coat paint. That is why we have to evaluate the sol-gel coating behavior in this configuration, because without sol-gel, we have no adhesion at all. In this case, the sol-gel coating is really an adhesion promoter.

**Figure 6:**
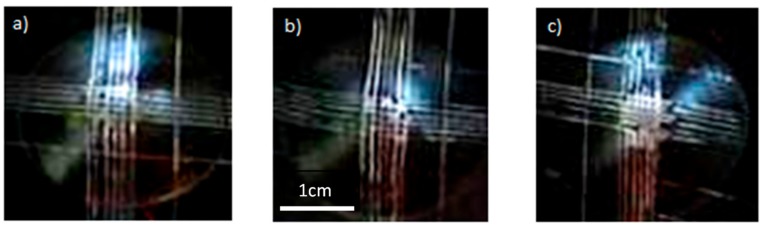
Shock test on (**a**) phosphated zinc + paint; (**b**) phosphated zinc + TEOS/MAP + paint; and (**c**) phosphated zinc + GPTMS/ASB + paint.

The shock test also highlights differences between the two formulations. As shown in Figure 6, no detachment of the coating system is observed for the formula TEOS/MAP (Figure 6b) compared to the GPTMS formulation (Figure 6c).

**Figure 7:**
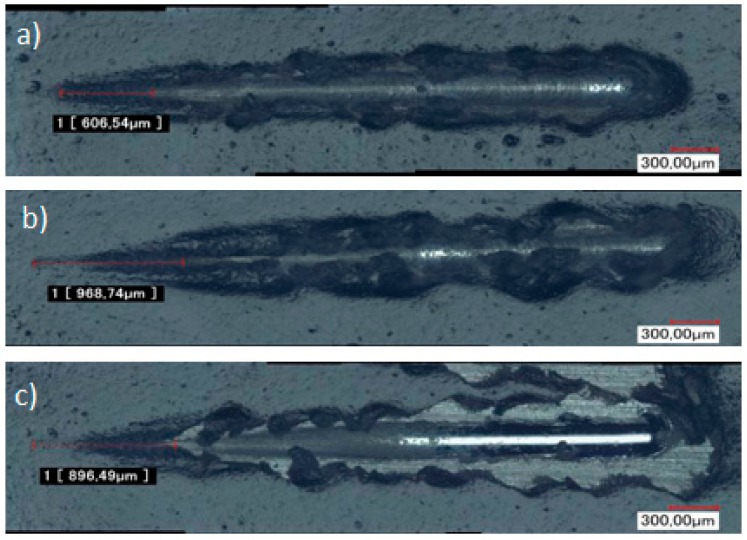
Optical micrographs of microscratch strip on (**a**) phosphated zinc + paint; (**b**) phosphated zinc + TEOS/MAP + paint; and (**c**) phosphated zinc + GPTMS/ASB + paint.

To investigate more deeply these adhesion properties, microscratch tests were carried out. The previous observations are confirmed at this micronic scale, since the delamination force is 9.68 N for TEOS/MAP and of 8.96 N for GPTMS/ASB. In addition, the profile for GPTMS/ASB reveals a lateral detachment of the coating, as observed in Figure 7.

To sum up these results, the TEOS/MAP-based coating combines both the best performances in adhesion and corrosion resistance.

Now, in order to further strengthen the corrosion resistance of the complete system, the organic precursor MAP was substituted for UPS in different ratios. This new precursor could improve the adhesion between the sol-gel coating and the top-coat paint.

### 2.2. TEOS/(MAP + UPS) Coating

As seen in the literature [21,22], UPS precursor is a good adhesion promoter due to the functional group (NH-CO-NH_2_). UPS is a hybrid precursor (organic-inorganic groups), and during the sol-gel network formation, covalent bonds are created, improving adhesion properties. Therefore, in order to increase TEOS/MAP coating performance, UPS is partially substituted in for the MAP. Five solutions corresponding to different molar substitution ratios of MAP by UPS (Table 1) were evaluated.

**Figure 8:**
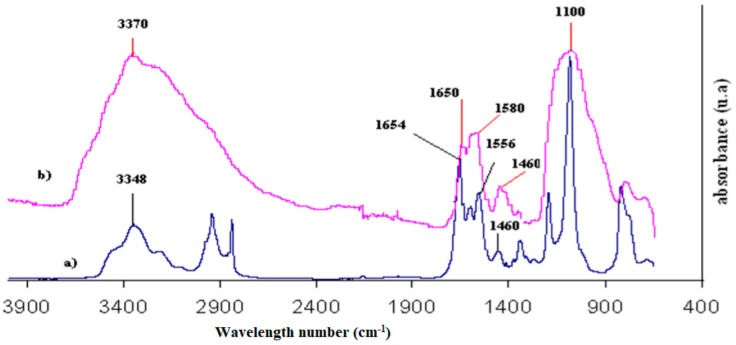
FTIR spectrum of (**a**) pure UPS and (**b**) TEOS/UPS coating on natural zinc.

Figure 8 displays the IR spectra of the UPS precursor (a) and the TEOS/UPS (b) coated natural zinc. The fine bands at 1200, 1110 and 810 cm^−1^ of the UPS precursor, corresponding to ν_SiC_, ν_SiOSi_ and δ_SiO_, respectively, are replaced by the footprint of the silicated matrix characterized by a broad band centered at 1100 cm^−1^ with a shoulder at 978 cm^−1^ and a weak band at 800 cm^−1^. The presence of the ureido function in the sol-gel network is demonstrated with the bands at 1650, 1610, 1560 and 1460 cm^−1^ related to ν_CO_, δ_NH2_ δ_CNH_ and δ_NH_, respectively. These four bands are also present in UPS precursor spectrum, indicating that the ureido functional group is unchanged after the network sol-gel formation, remaining available for adhesion with further painting-recovering. In the sol-gel coating spectrum, the NH, CH_2_ and CH_3_ stretching vibrations at 3348 and 2960 and 2840 cm^−1^ cannot be detected in the coated sample due to the broad band centered at 3370 cm^−1^, which corresponds to νOH of alcohol or adsorbed water. These results are in agreement with ureido grafted on silica [21] or periodic mesoporous organosilica [22], as already reported.

**Figure 9:**
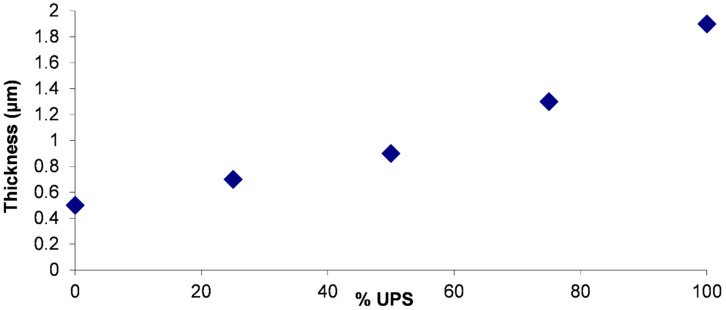
Evolution of the coating thickness according to the quantity of UPS.

As the UPS is a compound with high viscosity, it is important to check that its introduction into the sol-gel solution does not change its viscosity. The values are between 4.2 mPa.s and 4.6 mPa.s for the five tested formulations for shearing rates ranging between 644 and 699 s^−1^ at 23 °C. This small viscosity variation corresponds to the slight increase of the layer weight, which varies from 10.2 mg/dm^2^ to 11.3 mg/dm^2^ with UPS increasing quantity. The consequence is an increase in the coating thickness from 0.5 µm to 1.9 µm for 0% and 100% ureido, respectively (Figure 9). Note that between these two samples (without UPS and with 100% UPS), the coating thickness is almost increased by a factor of 4, while the weight of the layer is quite stable. This is probably due to the structuring role of the UPS compound in the deposit providing a different network of organic chains forming the coating.

**Figure 10:**
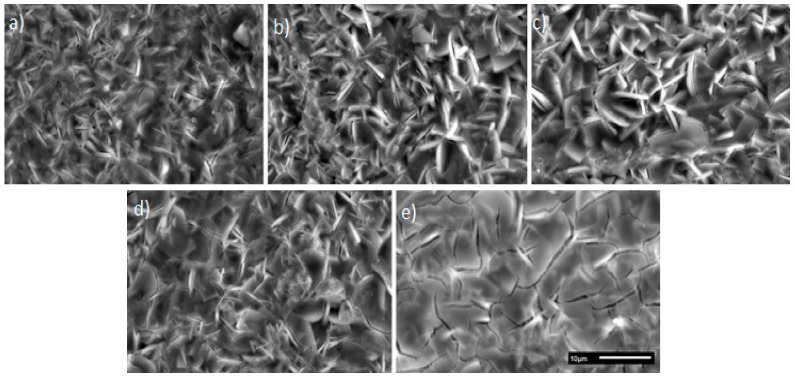
Scanning electron micrographs of coatings surface on phosphated zinc: (**a**) TEOS/MAP; (**b**) TEOS/(75%MAP + 25%UPS); (**c**) TEOS/(50%MAP + 50%UPS); (**d**) TEOS/(25%MAP + 75%UPS); and (**e**) TEOS/UPS (same scale bar of 10 µm for all images).

The deposits containing 0% and 25% of UPS are too thin (0.5 µm and 0.7 µm respectively) and do not properly cover the crystals of studied substrate (2 µm roughness). SEM observations of these 2 coatings revealed areas without any deposit (Figure 10a,b). These areas may weaken the integrity of the coating and give poor resistance against corrosion, so these 2 formulations were not selected for the following studies.

**Figure 11:**
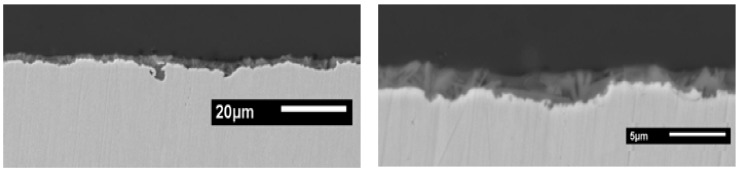
Scanning electron micrographs of cross-section of TEOS/(50%MAP + 50%UPS) coating deposited on phosphated zinc.

Interestingly, for the formulation with 50% of UPS, despite the thickness being only 0.9 μm, the entire surface is covered (Figure 10c and Figure 11). The coating follows the topography of the surface and creates a continuous layer above the crystals.

In contrast, for the 100% UPS coating, the presence of cracks in the coating is observable on SEM micrographs (Figure 9e). This is explained by the too large thickness and non-optimal drying conditions for this formulation. These cracks are defects in the coating that can degrade the resistance against corrosion, as mentioned and observed in the samples with thicknesses that were too low from the formulations containing a small amount of or no UPS (0–25% UPS). Based on these first surface observations, formulations with 0%, 25% and 100% of UPS were not selected for further investigation (corrosion and adhesion tests).

**Figure 12:**
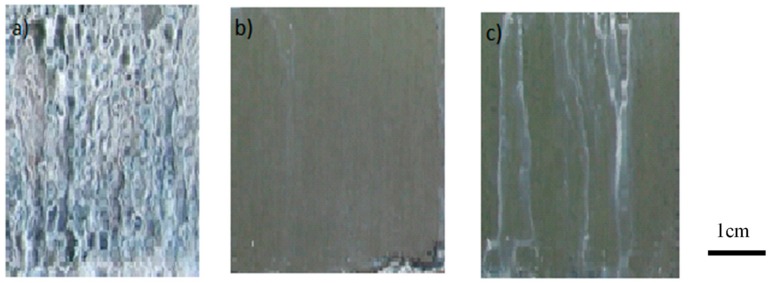
Salt spray test performed for 240 h on (**a**) phosphated zinc; (**b**) phosphated zinc + TEOS/(50%MAP + 50% UPS) coating; and (**c**) phosphated zinc + TEOS/(25%MAP + 75% UPS).

The climate chamber test did not discriminate between the 2 formulations tested (50% UPS and 75% UPS); after the 10 cycles, the two experimental conditions showed very few surface degradations, and the evolutions of mass gains were similar. On the other hand, the salt spray test proved more useful, since some differences appeared after 240 h of exposure (Figure 12). The addition of UPS significantly improves the corrosion resistance under salt spray conditions. Previously, slight degradations began to appear after 96 h of testing for the coating TEOS/MAP without UPS; whereas with 50% of UPS, the first degradations appeared after 168 h of testing. Significant differences under salt spray conditions appeared after 10 days (=240 h) of testing for the 2 coatings tested with 50% and 75% of UPS. The best performances in the salt spray test were obtained for the formulation TEOS/(50%MAP + 50%UPS).

**Figure 13:**
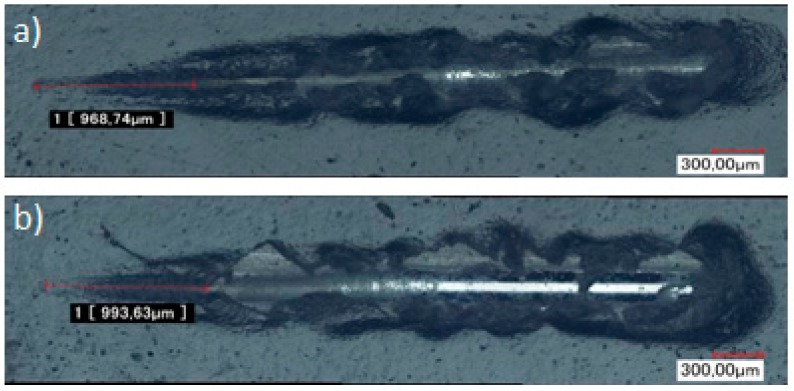
Optical micrographs of microscratch performed on (**a**) phosphated zinc + TEOS/MAP + paint; and (**b**) phosphated zinc + TEOS/(50%MAP + 50%UPS) + paint.

To ensure the UPS does not degrade the adhesion performance achieved with TEOS/MAP coating, shock and microscratch tests were conducted. As indicated before, paint was applied to the sol-gel samples in order to evaluate the adhesion properties of system. In this case, the comparison of the coating behavior between TEOS/MAP and TEOS/(50% MAP + 50% UPS) shows no difference for the shock test, and the results obtained in the microscratch test show a very slight difference when UPS is added to the formulation (critical load of 9.68 N for the sample coated with TEOS/MAP against 9.94 N for the sample with 50% UPS) (Figure 13).

The key point is that, in both cases, there is no degradation of the adhesion of the coating on the substrate, as previously observed with GPTMS/ASB-based coating.

So, this study confirmed that an organic–inorganic sol-gel hybrid formulation such as TEOS/MAP system was adequate for the corrosion inhibition of zinc substrates without degrading adhesion properties.

However, this study also revealed that the corrosion inhibition of coatings based on a ternary mixture of TEOS/MAP/UPS precursors could be further improved compared to TEOS/MAP systems.

As previously stated, the corrosion inhibition of sol-gel coatings is strongly affected by their structure, depending on the progress of hydrolysis and condensation reactions, forming the final sol-gel network. Even if promising formulations including alkoxysilanes have increased the corrosion inhibition of metallic substrates, the improved corrosion inhibition coatings including UPS precursor can be attributed to the formation of a denser and more compact sol-gel network and also to its higher degree of hydrophobicity [19,20]. This characteristic was checked by surface energy measurements, and hydrophobicity is well known to be important, because it prevents the access of water to the metal-coating interface.

On zinc substrate, an optimized formulation TEOS/(50% MAP + 50% UPS) was proposed, and the good performances were attributed to the formation of more compact and impermeable coatings due to the more extensive cross-linking between the reactive alkoxy groups of alkoxysilanes and UPS molecules.

The salt-spray tests showed that this formulation was the most effective for reaching the required industrial performances.

## 3. Materials and Methods

### 3.1. Materials and Surface Preparation

Table 2 gives the chemical composition of the zinc substrate in accordance with EN/DIN 1179 on which the chemical conversion treatment was done.

The dimensions of the samples are 40 mm × 100 mm × 0.7 mm thick and the 100 mm length corresponds to the rolling direction. Before deposition, surface preparation is necessary to obtain a clean surface in order to have a good homogeneity of the deposits. Samples are washed with ethanol and dried in pressurized air. Results presented in this paper are performed on at least 2 samples to ensure reproducibility of the tests.

### 3.2. Sol Preparation and Coating Process

Nitric acid (69%) was obtained from VWR, whereas all the precursors were purchased from Aldrich. Cerium nitrate (Ce(NO_3_)_3_, 6H_2_O) was added to each sol-gel formulation at the optimized concentration of 0.01 mol L^−1^.

The two formulations of hybrid coatings were developed in the CIRIMAT laboratory in order to protect different substrates, such as aluminum [17], zinc [16] or steel [18].

On one hand, a TEOS/MAP sol formulation is prepared starting from the mixture of tetraethylorthosilicate (TEOS) and 3(trimethoxysilyl)propyl methacrylate (MAP) with the optimized conditions: R = [TEOS]/[MAP] = 8; C = [TEOS] = 1 mol/L; H = [H_2_O]/([TEOS] + [MAP]) = 7. After the hydrolysis of these precursors, sol-gel reaction (hydrolysis and condensation) occurs before the cerium nitrate addition to ensure the role of corrosion inhibitor in the sol-gel matrix [23].

In this paper, the modification is relative to a substitution of MAP by (1-[3-(trimethoxysilyl)propyl]ureido (UPS) in order to simultaneously improve corrosion and adhesion properties.

On the other hand, a formulation based on 3-glycidoxypropyltrimethoxysilane (GPTMS) and aluminum tri-sec butoxide (ASB) as chemical precursors is similarly developed [16,17]. Cerium nitrate is dissolved in an alcoholic solvent (ethanol for TEOS/MAP and propanol for GPTMS/ASB), then organic and inorganic precursors are introduced with stirring and finally the demineralized water is added. After 10 min of stirring, pH is adjusted to a value of 3 with nitric acid. After a maturation of 24 h, protective films are deposited by dip-coating with a withdrawal speed corresponding to 20 cm/min with a Concoat DC2000 dip-coater.

Thereafter, the samples are dried for 15 min at 60 °C and 30 min at 180 °C in a drying oven for the TEOS/MAP and the GPTMS/ASB formulations, respectively.

### 3.3. Characterization Techniques

The sol viscosities were measured with a Rheomat RM100 rheometer for shearing rates ranging between 644 and 699 s^−1^. The samples are first visually examined before analyzing the microstructure of the coatings by Scanning Electron Microscope JEOL JSM–6510 L apparatus operating at 20 kV with a distance of 10 mm. Infrared spectrometry was performed by a Nicolet 6700 FT-IR Spectrometer from Thermo Scientific in ATR diamond mode directly on zinc substrate with the coating. Coatings are directly analyzed on natural zinc in order to get rid of the layer of phosphates which can interfere with the coating signal. Different peaks are analyzed in order to follow the formation of specific bonds in the coating.

The anti-corrosive barrier properties of the coatings were evaluated by exposure in salt spray until total surface degradation, and in a climatic chamber for a duration of 10 days. Salt spray presents corrosive conditions that are more aggressive than those in a climatic chamber; a first choice of coating formulation will be made according to the results in salt spray, and then a finer discrimination will be made following the test in climatic chamber.

The salt spray is an Ascoot S120XP model and test conditions are 100% wet atmosphere at 35 °C +/−2 °C with solution of 50+/−5 g/L of NaCl, pH between 6.5 and 7.2, atmospheric pressure between 0.7 and 1.4 bar and a flow in the chamber between 1 and 2 mL/h (ASTM B117). Before being placed in a salt spray chamber, the samples are prepared by masking the edges in order to prevent the spread of corrosion to the sides except on one side. Samples are visually observed at regular time intervals, every 24 h. A duration of 168 h of exposure without any full-face degradation of the coating (corrosion puncture) seems to be a suitable performance or criterion for sol-gel coatings applied on pre-patinated zinc.

The climatic chamber is a Q-fog cct600 and the conditions of the test are presented in Table 3 with alternating dry and wet steps. Samples are observed and weighed every 24 h in order to evaluate the weight gain corresponding to the formation of rust due to corrosion. Indeed, because the zinc corrosion products are adherent to the substrate, the weight gain measured during the climatic chamber test corresponds to the formation of white rust and quantifies the corrosion resistance performance of the coating.

The adhesion properties were evaluated by shock tests (ISO 6272) and scratch tests. The first one is an industrial test, and the device used is a TQC IP 2000; the samples are squared with a multi-blade cutter to weaken the lacquer at the start and after deformation, the number of non-adherent coating squares is noted and a quotation is given to the sample according to ISO 2409. Scratch tests were performed using a scratch-test revetest instrument (CSM Instruments) with a diamond indenter with conical geometry. The scratch test was carried out with a progressive loading from 0 to 40 N, at a scanning speed of 10 N/min. After the scratch test, the mechanism of scratch delamination and the critical loads were visualized by optical microscopy. The critical load is the force determined when the delamination phenomenon appears.

## 4. Conclusions

The results proved that phosphated zinc surface pre-treatments by silane-based coatings are very promising for developing multifunctional coatings exhibiting both corrosion resistance and adhesion strength. These performances can be attributed to the presence of the organic-inorganic functional groups in the sol-gel network. Strong covalent bonds can improve the adhesion properties as well as the barrier effect to protect the substrate against corrosion.

Several surface mechanical investigations, topographic analyses, salt-spray tests and microstructural characterizations have been performed to determine the more adequate formulation.

Among the numerous formulations investigated and the varied synthesis parameters, it has been proved that the best compromise for achieving both properties (anti-corrosion and adhesion) and a high durability level is a TEOS-MAP sol-gel system in which 50% of MAP precursor is replaced by UPS.

The next step will be to transfer and implement such formulations at a pilot scale on industrial sites.

## Figures and Tables

**Table 1:** Composition of the TEOS/(MAP + UPS) formulations.

	Molar Ratio	
	% MAP	% UPS
TEOS/MAP	100	-
TEOS/(75%MAP + 25%UPS)	75	25
TEOS/(50%MAP + 50%UPS)	50	50
TEOS/(25%MAP + 75%UPS)	25	75
TEOS/UPS	-	100

**Table 2:** Chemical composition of zinc alloy.

Element	Zinc	Titanium	Copper	Aluminum
% mass	balance	0.06 to 0.20	0.08 to 1	0 to 0.015

**Table 3:** Test conditions in climatic chamber.

Step	Atmosphere	Temperature	Duration
1	Humid	60 °C	4 h
2	dry	35 °C	1 h
3	Humid	60 °C	4 h
4	dry	35 °C	1 h
5	Humid	60 °C	6 h
6	dry	35 °C	2 h
7	Humid	60 °C	3 h
8	dry	55 °C	3 h

## References

[1] Liu Y.W., Wang Z.Y., Cao G.W., Cao Y., Huo Y. (2017). Study on corrosion behavior of zinc exposed in coastal-industrial atmospheric environment. Mater. Chem. Phys..

[2] Duran A., Castro Y., Aparicio M., Conde A., De Damborenea J.J. (2007). Protection and surface modification of metals with sol-gel coatings. Int. Mater. Rev..

[3] Wang D., Bierwagen G.P. (2009). Sol-gel coatings on metals for corrosion protection. Prog. Org. Coat..

[4] Figueira R.B., Silva C.J.R., Pereira E.V. (2015). Organic–inorganic hybrid sol–gel coatings for metal corrosion protection: A review of recent progress. J. Coat. Technol. Res..

[5] Van Ooij W.J. (2005). Corrosion Protection Properties of Organofunctional Silanes—An Overview. Tsinghua Sci. Technol..

[6] Osborne J.H. (2001). Observations on Chromate Conversion Coatings from a Sol–Gel Perspective. Prog. Org. Coat..

[7] Metroke T.L., Parkhill R.L., Knobbe E.T. (2001). Passivation of Metal Alloys Using Sol–Gel-Derived Materials—A Review. Prog. Org. Coat..

[8] Eliziane M., Ariza E., Baleester M., Pagotto I.V., Rocha L.A., De Alvarenga C.M. (2006). Characterization of Organic–Inorganic Hybrid Coatings for Corrosion Protection of Galvanized Steel and Electroplated ZnFe Steel. Mater. Res..

[9] Singh A.K., Rout T., Narayan R., Verma A.K., Bandyopadhayay N., Rani N. (2010). Anti Corrosion Sol-Gel Hybrid Coating on Zinc and Zinc Alloy Steel Sheets and Preparing Method Thereof. India Patent.

[10] Singh A.K., Rout T., Narayan R., Verma A.K., Bandyopadhayay N., Rani N. (2011). Anticorrosive Hybrid Sol-Gel Film on Metallic Substrates and Method of Producing the Same. U.S. Patent.

[11] Volentiru E., Nyari M., Szabo G., Horvolgyi Z., Muresan L.M. (2014). Silica sol-gel protective coatings against corrosion of zinc substrates. Per. Pol. Chem. Eng..

[12] Ali Shimaa M., Al lehaibi Hamedh A. (2017). Protective sol-gel coatings for zinc corrosion: Precursor type effect. Surf. Coat. Technol..

[13] Garcia-Heras M., Jimenez-Morales A., Casal B., Galvan J.C., Radzki S., Villegas M.A. (2004). Preparation and electrochemical study of cerium–silica sol–gel thin films. J. Alloys Compd..

[14] Bibber J.W. (2008). Non-chrome-Containing Conversion Coatings for Zinc and Zinc Alloys: Environmentally Friendly Alternatives Provide Equal or Better Adhesion and Corrosion Resistance as Conventional Methods. Met. Finish..

[15] Fedel M., Olivier M., Poelman M., Deflorian F., Rossi S., Druart M.-E. (2009). Corrosion protection properties of silane pre-treated powder coated galvanised steel. Prog. Org. Coat..

[16] Meiffren V., Dumont K., Lenormand P., Manov S. (2011). Development of new processes to protect zinc against corrosion, suitable for on-site use. Prog. Org. Coat..

[17] Cambon J.B., Esteban J., Ansart F., Bonino J.P., Turq V., Santanieli S.H., Santilli C.V., Pulcinelli S.H. (2012). Effect of cerium on structure modifications of a hybrid sol–gel coating, its mechanical properties and anti-corrosion behavior. Mater. Res. Bull..

[18] Cambon J.B., Ansart F., Bonino J.P., Turq V. (2012). Effect of cerium concentration on corrosion resistance and polymerization of hybrid sol-gel coating on martensitic stainless steel. Prog. Org. Coat..

[19] Fir M., Orel B., Šurca Vuk A., Vilčnik A., Ješe R., Francetič V. (2007). Corrosion Studies and Interfacial Bonding of Urea/Poly(dimethylsiloxane) Sol/Gel Hydrophobic Coatings on AA 2024 Aluminum Alloy. Langmuir.

[20] Šurca Vuk A., Fir M., Ješe R., Vilčnik A., Orel B. (2008). Structural studies of sol–gel urea/polydimethylsiloxane barrier coatings and improvement of their corrosion inhibition by addition of various alkoxysilanes. Prog. Org. Coat..

[21] Cousinié S., Gressier M., Alphonse P., Menu M.J. (2007). Silica-Based Nanohybrids Containing Dipyridine, Urethan, or Urea Derivatives. Chem. Mater..

[22] Wei Q., Liu L., Nie Z.R., Chen H.Q., Wang Y.L., Li Q.Y., Zou J.X. (2007). Functionalization of periodic mesoporous organosilica with ureidopropyl groups by a direct synthesis method. Microporous Mesoporous Mater..

[23] Certhoux E., Ansart F., Turq V., Bonino J.P., Sobrino J.M., Garcia J., Reby J. (2013). New sol-gel formulations to increase the barrier effect of a protective coating against the corrosion of steels. Prog. Org. Coat..

